# Lolitrem B and Indole Diterpene Alkaloids Produced by Endophytic Fungi of the Genus *Epichloë* and Their Toxic Effects in Livestock

**DOI:** 10.3390/toxins8020047

**Published:** 2016-02-15

**Authors:** Guerre Philippe

**Affiliations:** Université de Toulouse, INP, ENVT, UR Mycotoxicologie, F-31076 Toulouse, France; p.guerre@envt.fr; Tel.: +33-056-119-3840

**Keywords:** alkaloids, endophytic fungi, *Epichloë*, lolitrem B, mycotoxins, livestock, toxicology, staggers

## Abstract

Different group of alkaloids are produced during the symbiotic development of fungal endophytes of the genus *Epichloë* in grass. The structure and toxicity of the compounds vary considerably in mammalian herbivores and in crop pests. Alkaloids of the indole-diterpene group, of which lolitrem B is the most toxic, were first characterized in endophyte-infected perennial ryegrass, and are responsible for “ryegrass staggers.” Ergot alkaloids, of which ergovaline is the most abundant ergopeptide alkaloid produced, are also found in ryegrass, but generally at a lower rate than lolitrem B. Other alkaloids such as lolines and peramine are toxic for crop pests but have weak toxicological properties in mammals. The purpose of this review is to present indole-diterpene alkaloids produced in endophyte infected ryegrass from the first characterization of ryegrass staggers to the determination of the toxicokinetics of lolitrem B and of their mechanism of action in mammals, focusing on the different factors that could explain the worldwide distribution of the disease. Other indole diterpene alkaloids than lolitrem B that can be found in *Epichloë* infected ryegrass, and their tremorgenic properties, are presented in the last section of this review.

## 1. Introduction

**Figure 1:**
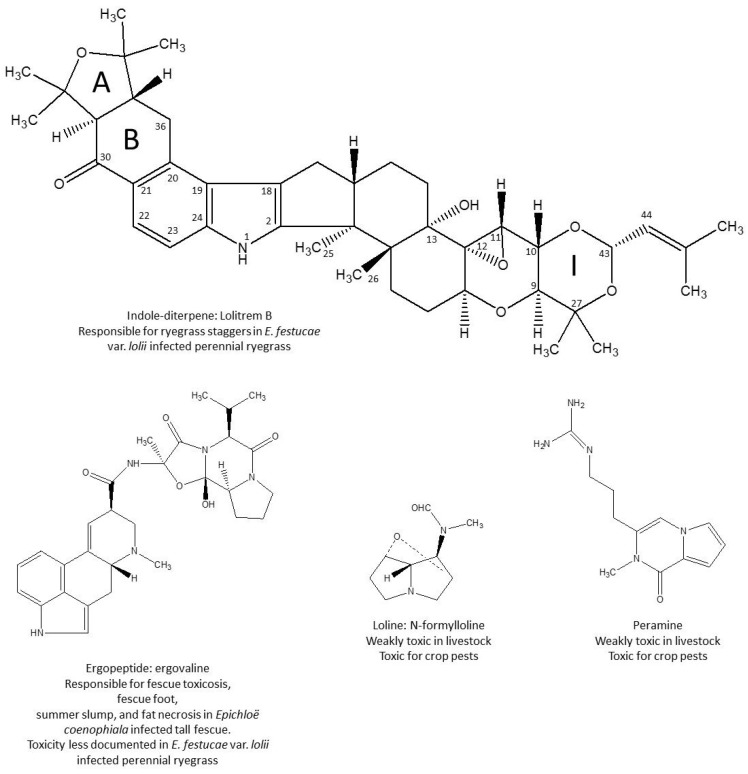
Main alkaloids produced by *Epichloë* in perennial ryegrass.

The symbiotic development of fungal endophytes of the genus *Epichloë* in grass results in the production of different groups of alkaloids (Figure 1) whose profile of production in plants explains signs of toxicity. Lolitrem B was recognized as the main indole-diterpene alkaloid produced in *Lolium perenne* (perennial ryegrass) infected by *E. festucae* var. *lolii* (*Neotyphodium* *lolii)*, responsible for “ryegrass staggers” in livestock [1,2]. Other indole-diterpene alkaloids have been characterized in endophyte infected ryegrass, but differ from lolitrem B in their tremorgenic properties [3]. Ergot alkaloids have been found in *L. perenne*, in *L. arundinaceum* (tall fescue), and in other grasses [4,5]. Ergovaline has been recognized as the most abundant and the most toxic ergopeptide alkaloid produced in *E. coenophiala*-infected tall fescue, and is responsible for fescue toxicosis in livestock [5]. By contrast, in *E. festucae* var. *lolii*-infected perennial ryegrass, the signs of ergot alkaloid toxicity are often masked by tetanic spams and staggers, which are linked to the ingestion of lolitrem B [6]. Other alkaloids produced in endophyte-infected perennial ryegrass include lolines, of which *N*-formylloline is the most abundant, and peramine, which is well tolerated by livestock but toxic for crop pests [6,7].

Because the alkaloids produced by the fungal endophyte are responsible for serious diseases and economic losses in livestock, a simple solution to avoid toxicity could be to eliminate the endophyte from the grass. However, alkaloids are also toxic for insects and nematodes and the cultivars that are free of endophytes are more sensitive to crop pests than the corresponding endophyte-infected cultivars. So, most recent research and development on endophyte-infected grasses has focused on the selection of *Epichloë* strains that are unable to produce the alkaloids that are highly toxic in livestock, *i.e.*, ergovaline and lolitrem B, but still able to produce the alkaloids that are toxic for insects and nematodes [6]. On the other hand, non-selected endophytes (also known as “wild” endophytes) are still present in grasses in several countries, and questions persist concerning the production of alkaloids in plants and their toxicity in livestock. The purpose of this review is to present the indole diterpene alkaloids that are produced by endophytic fungi of the genus *Epichloë* and their toxic effects in livestock, focusing on lolitrem B and on the factors responsible for variations in its level in grasses and in its toxicity in livestock.

## 2. Ryegrass Staggers, from Its First Characterization to the Discovery of Lolitrem B

The first description of muscular incoordination in cattle and horses grazing on ryegrass was made in New Zealand in 1906. Because the disease was observed when seeds were formed, the sclerotia of *Claviceps purpurea* were considered to be causative agents until the 1950s. During the same period, a fungus was found to infect *L. perenne* seeds in different countries [8]. The fungus was called “endophyte” because the mycelium developed inside the plant cells and invaded the whole plant except the roots. It was seed transmitted, but apparently not transmitted by the pollen [9]. In 1935, a review on “staggers” in livestock pointed to the different etiology of the syndrome: a metabolic origin of the signs in cows after parturition, a sclerotia origin in some cases of *Paspalum* staggers, whereas there was some doubt about the sclerotia origin in at least some cases of ryegrass staggers, especially because the disease occurred when the grass was particularly short, after a period of dryness, in the absence of seeding [10]. However, feeding endophyte-infected seeds to mice, rat, chickens, and sheep failed to reveal deleterious effects, and the toxicity of the fungal endophyte remained unheeded for several years [11]. Although the etiologic agent of ryegrass staggers was unknown, it was clear that the disease occurred in sheep grazing extremely short grass [12], but experimental reproduction of the disease by feeding sheep with ergots of *C. purpurea* failed to produce symptoms of ryegrass staggers [13]. By contrast, staggers were experimentally produced in sheep grazing the base of the ryegrass plant, whereas no signs were observed in sheep that were prevented from grazing this part of the plant [14]. Penitrem A, verruculogen, fumitremorgin B, and paxilline, which are tremorgenic mycotoxins of different fungal origin, were suspected to be the causative agent of ryegrass staggers [15]. It was hypothesized that mycotoxins are produced in the soil by saprophytic fungi, then translocated to the plant via the ryegrass roots [16]. In 1981, a feeding trial conducted to compare the rate and severity of staggers in sheep with the rate of endophyte infection of ryegrass definitively confirmed the implication of the endophyte [1]. Lolitrem A and B were the main mycotoxins isolated in endophyte-infected perennial ryegrass [2], whereas feeding sheep with seeds that contained lolitrems made it possible to reproduce ryegrass staggers [17]. Consequently, lolitrem B produced by *E. festucae* var. *lolii* during its symbiotic development in *L. perenne* was considered to be responsible for ryegrass staggers. The toxic threshold was established as being between 1800 and 2000 µg lolitrem B/kg feed in cattle and sheep [18]. However, although other alkaloids than lolitrem B can be produced by *Epichloë* in endophyte-infected ryegrass, little is known about their effects during concomitant exposure to lolitrem B. This was the case for ergot alkaloids and ergovaline, for which signs of toxicity are rarely reported in livestock fed endophyte-infected ryegrass, whereas they are the main alkaloids involved in the toxicity of endophyte-infected tall fescue [6]. This difference was linked to the profile of alkaloid production by *Epichloë* in the infected plant [5]. The concentration of lolitrem B is usually 5 to 10 fold higher than ergovaline in endophyte-infected ryegrass [19,20,21,22,23], so most studies focused on lolitrem B, and little information was available on ergovaline in these plants. In New Zealand in particular, most of the toxicity of ergot alkaloids in endophyte-infected perennial ryegrass remained unknown until the development of a novel endophyte (known as “endosafe”) that was unable to produce lolitrem B [6]. Interestingly, various studies conducted in lambs and in lactating ewes also suggested that the toxic threshold of ergovaline was lower in endophyte-infected ryegrass than in endophyte-infected tall fescue [24,25,26]. By contrast, a synergistic effect between ergotamine and lolitrem B was observed in smooth muscle contractile tension in longitudinal preparation of the distal colon in sheep [27]. It was suggested that this effect could contribute to the more prevalent rate of noninfectious diarrhea, observed in sheep grazing endophyte-infected pastures [27]. Interaction between lolitrem B and ergovaline could also contribute to decreases in milk production observed in dairy cows grazing on endophyte-infected ryegrass [28,29,30,31]. In the same way, little is known about the production and toxicity of indole-diterpene alkaloids other than lolitrem B, including the intermediate metabolites of its synthesis by *Epichloë*.

## 3. Lolitrem B, a Tremorgenic Mycotoxin

**Figure 2:**
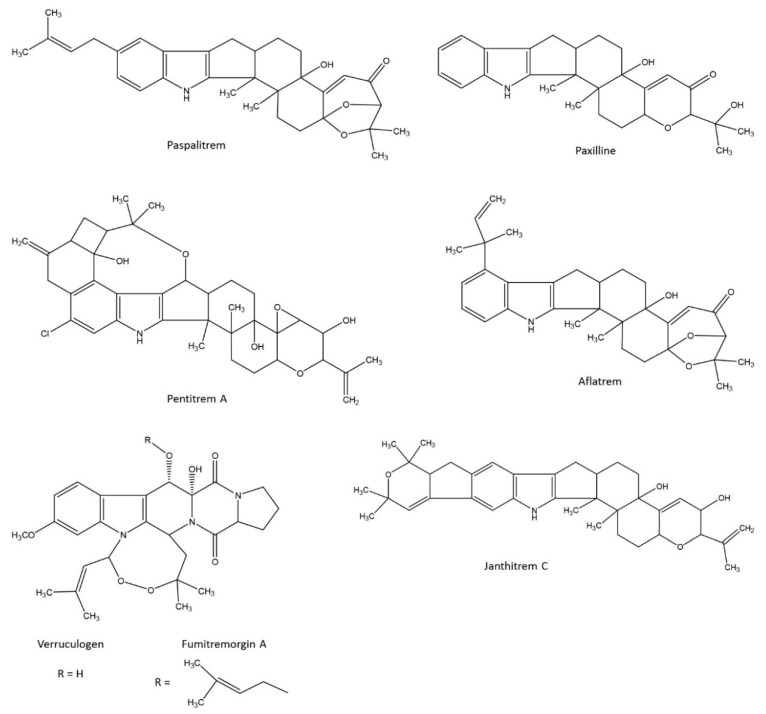
Structures of some tremorgenic mycotoxins.

Signs of staggers are commonly observed in animals fed with mycotoxins such as paspalitrem, paxilline, penitrem, aflatrem, verruculogen, fumitremorgin, janthitrem, and lolitrem (Figure 2). Paspalitrems are produced by different species of the genus *Claviceps* that parasitize the seeds of *Paspalum* grasses, Bermuda grass, and other grasses [32]. Paxilline, penitrem, aflatrem, verruculogen, fumitremorgin, and janthithrem are produced by fungi of the genera *Aspergillus* or *Penicillium*, most of which are saprophytic in cereals and plants during storage, and some are also phytopathogenic [33]. Paxilline, epoxy-janthitrem, and lolitrem are produced by endophytic fungi of the genus *Epichloë* during their symbiotic development in some grasses [34]. Although these compounds are of different fungal origin, several studies were conducted to compare their mechanism of action in the course of elucidation of lolitrem B mechanism of action. Both the toxicokinetic and pharmacological properties of tremorgenic mycotoxins can vary in terms of the location (peripheral *vs.* central), nature (excitatory *vs.* inhibitory), and severity (amplitude, duration) of the effects.

### 3.1. From Staggers to BK-Channels

The term “staggers” is used to describe a variety of diseases in livestock whose etiology can differ considerably, ryegrass ingestion being only one possible disease etiology [10]. Staggers themselves vary in both morbidity and severity during the course of ryegrass toxicity. From 5% to 75% of the animals in the same herd are affected, and signs vary from slight trembling of the neck after hard exercise to severe tetanic spasm and collapse, a system of scoring being used to assess the severity of the disease [12,14,35]. Until the discovery of lolitrems in 1981, several tremorgenic mycotoxins were suspected to be the causative agent of ryegrass staggers [2,15] and various *in vivo* and *ex vivo* experiments were conducted to understand their mechanism of action.

Studies using sheep and rat synaptosomes revealed that verruculogen and penitrem A act by interfering with the release of amino acid neurotransmitters. The tremors could be due to anomalous release of both excitatory and inhibitory transmitters at central and peripheral synapses, leading to loss of coordination of the neural mechanisms that control muscle action [36]. Enhanced unstimulated release of the excitatory amino acid neurotransmitters aspartic acid and glutamic acid was measured in cerebrocortical synaptosomes prepared from sheep showing severe symptoms of ryegrass staggers [37]. Studies in rat brain membranes revealed that the four tremorgenic mycotoxins tested (aflatrem, paspalinine, paxilline, and verruculogen) inhibited GABA-induced ^38^CI^−^ influx into rat brain microsacs, while a non-tremorgenic mycotoxin, verruculotoxin, had no effect [38]. It was suggested that the relatively nonpolar properties of these mycotoxins enable them to pass the blood-brain barrier and gain rapid access to many of the synapses present in the brain, where they have a central effect [36].

A bioassay was developed in mice to assess the tremorgenic properties of mycotoxins [39]. After intraperitoneal administration, lolitrem B was responsible for tremors whose onset was slower but lasted longer than those caused by aflatrem [40]. Lolitrem B also had a much longer and more potent tremorgenic effect than paxilline [41]. Electromyographic activity of skeletal muscle recorded in sheep receiving different levels of penitrem, paxilline, and lolitrem B revealed that the excitatory properties of all the compounds agree with the previously observed effect [42].

The effects of tremorgenic mycotoxins were also investigated on smooth muscles, but were more difficult to interpret. Verruculogen, penitrem B, and paxilline enhanced the electrically-stimulated contractions of guinea pig ileum but did not influence the contractions caused by exogenous acetylcholine, suggesting that these compounds enhance the release of acetylcholine [43]. Penitrem, paxilline, and lolitrem B induced variable responses of the smooth muscle of the reticulorumen, abomasum, and duodenum in sheep [44]. An inhibitory effect on the abomasum was observed whereas both excitatory and inhibitory effects were observed on the duodenum [42]. By contrast, an excitatory effect, which was partially blocked by atropine, was observed on the reticulorumen [45]. The different responses of the gastro-intestinal tract to lolitrem B in sheep could be partly explained by interference in the release of acetylcholine by the parasympathetic nerve, which is required for the cyclical contractions of the reticulorumen, but does not occur in the abomasum and duodenum [46].

Sarcolemmal membrane vesicles of bovine aortic smooth muscle were used to compare the properties of lolitrem B and paspalicine, a non-tremorgenic analog of paspalinine that is dehydroxylated [47]. Because both compounds blocked large conductance calcium-activated potassium channels (BK channels), it was concluded that although some of the pharmacological properties of lolitrem B can be explained by inhibition of BK channels, tremorgenicity may not be related to this mechanism of action [47]. Twenty years after the first discovery of lolitrem B, toxic thresholds have been established for the occurrence of staggers [18], but its pharmacological mechanism of action remains unclear as both peripheral and central effects are suspected.

Knowledge of the mechanism of action of lolitrem B at the pharmacological level advanced considerably when paxilline and lolitrem B toxicity were compared in wild mice and in knock-out mice deficient in BK channels (Kcnma1). BK channels are expressed in cell membranes of all the tissues where they enable outflow of K^+^, which is responsible for hyperpolarization of the cells and a reduction in cellular activity [48]. The fact that tremors occurred in wild mice but not in the mice lacking Kcnma1 demonstrated the important role of BK channels in rye grass staggers. The observation that known lethal doses of lolitrem B in the wild-type mice had no effect in knock-out mice also suggested that inhibition of BK channels by lolitrem B is probably the only mechanism of action involved in the acute toxicity of lolitrem B [49]. Relationship structure activities were performed on BK channels, making it possible to compare different structurally-related lolitrems [50]. This confirmed that only a small change in structure modifies the binding properties of the toxins, as previously observed for tremorgenicity in the mouse bioassay [51]. Comparison of paxilline and lolitrem B also revealed that lolitrem B is more potent than paxilline at inhibiting BK channels *in vitro* and that inhibition cannot be reversed by washing [52]. This result is in agreement with previous observations in *in vivo* studies in mouse, in which lolitrem B had a much longer and more potent effect on motor function than paxilline, whereas paxilline caused a more rapid onset of tremor, but tremors lasted for a shorter period than those caused by lolitrem B [41].

### 3.2. Toxicokinetics

Both central and peripheral effects of lolitrem B could contribute to toxicity; however, because lolitrem B is insoluble in aqueous media, it is difficult to obtain, and relatively little is known about its toxicokinetics. All the studies performed after oral dosing of lolitrem B revealed very low absorption of the toxin [31,53]. No study has reported lolitrem B concentrations in the brain, but 14C-labeled paxilline injected intraperitoneally in the mouse showed that the toxin was present in the brain but at extremely low concentrations [41]. After high doses of lolitrem B intravenously administered in sheep (75 µg/kg BW), the concentration of lolitrem B in serum decreased rapidly, whereas tremors continued for 16 hours post dosing [41]. Rapid elimination of lolitrem B from serum was confirmed in intravenously dosed (23 µg/kg BW) lactating goats, and a half-life of 14 min was calculated [53]. Both the rapid elimination of the toxin from serum and the time at which tremors occurred after IV dosing suggest that lolitrem B may be stored in a specific compartment of the body, then progressively released into the blood at very low levels for transport to the brain. This hypothesis was strengthened by analysis of lolitrem B levels in body fluids and tissues. In goats, lolitrem B was present in milk for 32 h after the intravenous injection (one dose of 23 µg/kg BW) with an excretion rate of 3%. This result was confirmed after oral dosing (one dose of 100 µg/kg BW) in lactating goats: the duration of elimination in milk was 75 h and the excretion rate of the administered dose was 0.19% [53]. A similar result was observed in dairy cows after prolonged exposure to the toxin, when only 0.23% of the lolitrem B consumed was excreted into the milk [31].

Because lolitrem B is very soluble in organic solvents and in lipid media, it was hypothesized that it could be stored in fat storage [41]. High levels of lolitrem B in fat were confirmed in all the experiments in which lolitrem B was measured, in both cattle and sheep, and in both growing and lactating animals [25,31,54,55,56]. When sheep grazed for a prolonged period in pastures that contained lolitrem B, the toxin concentration in the fat rapidly increased with an increase in the concentration in the pasture, and decreased with a decrease in the concentrations in the pasture [55]. This last result suggested that, rather than accumulating, the quantities in the fat respond rapidly to the amounts being consumed by the animals [55].

Both the time at which tremors are observed after IV dosing and the lipophilic nature of lolitrem B suggest that the toxin may be metabolized. However, very little is known about the biotransformation of lolitrem B or the interactions between the toxin and drug-metabolizing enzymes. A study conducted in lactating ewes fed with endophyte-infected perennial ryegrass hay containing lolitrem B and ergovaline revealed slight effects on the activities of some drug-metabolizing enzymes [25]. These results were difficult to interpret because feeding endophyte-infected tall fescue hay that contained ergovaline alone changed some drug metabolizing enzymes activities in this species at lower levels of exposure [24]. However, comparison of the results obtained in the two studies suggests that interaction between lolitrem B and ergovaline may affect the activities of drug-metabolizing enzymes [24,25]. Alternatively, the importance of cytochrome P450 in the biosynthesis of lolitrem B, and the role of the transformations catalyzed by these enzymes on the biological activity of the toxins are notable [57,58].

## 4. Lolitrem B in Plants and Ryegrass Staggers

### 4.1. Worldwide Distribution of the Disease

Whereas infection of *L. perenne* by *E. festucae* var. *lolii* is reported worldwide, most outbreaks of ryegrass staggers have been reported in Australia and New Zealand [59]. By contrast, only sporadic atypical cases are observed in Europe and America. Most cases reported in Europe involved one animal or a limited number of animals after consumption of hay. The disease has been diagnosed in horses, bulls cattle, dairy cows, and sheep following observation of the typical clinical signs of staggers [23,60,61,62,63]. The diagnosis was confirmed by demonstrating high lolitrem B concentrations in the suspect hay. Along the northern coast of California, some cases of staggers have been diagnosed in sheep and cattle with a history of ingestion of perennial ryegrass [64]. Staggers was also observed in Japan in cattle and horses fed with ryegrass straw imported from Oregon [65]. In South America, a typical outbreak of ryegrass staggers was observed in Argentina in about 50% of the heifers of a herd of 560 animals after grazing 26 days in a paddock of pure *L. perenne*. Microscopic examination of plants confirmed the presence of an endophytic fungus [66].

Several studies conducted in Australia and New Zealand have demonstrated that the rate of infestation of ryegrass generally correlates with the frequency and severity of staggers [59,67,68,69]. A high rate of endophyte infection in perennial ryegrass was selected by plant breeders because it enhances growth and persistence of ryegrass in the pasture. For example, in New Zealand, improved persistence of endophyte-infected ryegrass has been associated with improved resistance to the Argentine stem weevil (*Listronotis bonariensis*) [70]. Resistance to other pests has been demonstrated [71,72,73]. In Europe, the rate of *Epichloë* infestation of perennial ryegrass varies considerably, depending on the country and the geographical location of the plants concerned [74,75,76,77,78,79]. A relatively lower rate of infestation in Europe compared with Australasia could partly explain the lower prevalence of staggers, but the rate of infestation alone was probably not sufficient to explain all the differences. The genetics of the endophytes and other factors that change the level of lolitrem B in plant may contribute to differences in the prevalence of ryegrass staggers worldwide.

### 4.2. Lolitrem B Biosynthesis

Since lolitrem B was recognized as the main toxin responsible for staggers in endophyte-infected ryegrass, several studies have been conducted to determine the genetic factors responsible for its synthesis. Although a large number of endophyte strains have been identified in plants, perennial ryegrass has been found to be the host of a limited number of distinct endophyte taxa [80,81]. Genetic analysis of *E. festucae* var. *lolii* strains demonstrated that some strains synthesize ergovaline alone, and others synthesize lolitrem B alone, but most produce variable concentrations of both toxins [82,83]. Analysis of the genetic factors required for the production of lolitrem B revealed that 10 different genes are present in a complex locus (*ltm*) organized in three clusters interspersed with transposon relics [83,84]. Paspaline was proposed as the intermediate metabolite forming the structural backbone required for the production of more complex compounds such as lolitrems and terpendoles [58,80]. The different metabolites produced during lolitrem B synthesis and their properties with respect to staggers are reviewed in the following paragraph. Although it is clear that the lack of a gene involved in the synthesis of lolitrem B makes it possible to predict the lack of its synthesis and its absence in plants, the presence of *ltm* genes does not make it possible to predict their level of expression or the level of lolitrem B in pastures. Indeed, comparisons of lolitrem B concentrations in the whole plant revealed major variations depending on which part of the plant was analyzed and on the location of the study. Field studies in Australia revealed concentrations of lolitrem B ranging from 0 to 4.44 mg/kg in perennial ryegrass, and of more than 1.8 mg/kg in 37% of pastures [85]. Other studies of the straw of endophyte-infected perennial ryegrass in Oregon also revealed a wide range of lolitrem B concentrations, from 0 to more than 5 mg/kg [22].

Selection of *E. festucae* var. *lolii* strains in Australasia also revealed marked variations in the production of alkaloids [6,86]. Whereas wild strains produced lolitrem B, ergovaline, and peramine, the “endosafe,” “AR1,” and AR37” strains did not produce lolitrem B. Unfortunately, because the “endosafe” strain still produced ergovaline, unexpected cases of toxicity characterized by intolerance to heat stress were reported [6,87]. By contrast, the “AR1” strain only produced peramine, which is not toxic for mammalian herbivores, but this strain provides only moderate protection against crop pests [55,86,87]. Alkaloids produced by “AR37” are discussed in the following section. In Europe, the beneficial role of endophyte infection in plant persistence or resistance to stress and invertebrate is less well understood [88,89,90,91]. However, studies on different ecotypes have revealed that the origin/genetics of the ecotype influence the concentration of lolitrem B in plants [92,93]. Interestingly, comparison of lolitrem B concentrations in ecotypes in Germany and in New Zealand also revealed that the environmental conditions of growth (mainly nutrients and climatic factors, also known as abiotic factors) that occurred during plant growth have a stronger influence on the concentration of alkaloids in plants than the origin/genetics of the ecotype [92]. However, little is known about the influence of abiotic factors on the level of lolitrem B in plants.

### 4.3. Variations in Concentrations of Lolitrem B in Plants

Environmental conditions can affect the level of alkaloids in plants; differences depend on plant-endophyte genotype interactions and on the compounds produced [91]. Lolitrem B concentrations in endophyte-infected perennial ryegrass vary considerably depending on the ecotype studied but also on the time of year and the location of the study. In Australia and New Zealand, lolitrem B maxima are reached in March, which corresponds to fall in the northern hemisphere [85,94,95]. By contrast, in France and Germany, the highest concentrations of lolitrem B are observed in June to August, with some differences depending on the ecotypes analyzed [92,93,96]. The maxima were seen to range from 2 to 10 mg lolitrem B/kg in the whole plant [22,85,92,93,94,95,96]. Peak concentrations of the other alkaloids produced were generally reached at the same time as the peak in lolitrem B, although with some differences. The peak ergovaline concentration has generally been observed in spring during flowering [20,93,94,96,97,98]. Another ergovaline peak, corresponding to plant regrowth, has also been reported in the fall, and was sometimes higher than the peak in the spring, especially in Australasia [20,85,93,94,95,96,97,98].

The distribution of lolitrem B in the plant also varies depending on the part of the plant analyzed and the stage of maturation of the plant. A study conducted over two years in endophyte-infected perennial ryegrass in France revealed a concentration of lolitrem B in the base, leaves, and inflorescence ranging from 0.01 to 3 mg/kg dry matter [96]. Over the study period, the highest concentrations of lolitrem B were always observed at the fully ripe stage in the inflorescence, followed by the base of plants, while the leaves contained the lowest levels [96]. Similar results have been observed for ergovaline. Likewise, lolitrem B but not ergovaline was reported to accumulate in the base of the plant and in senescent tissues; the distribution of the endophyte did not play a major role in the distribution of alkaloids in the plant [99,100].

Nitrogen fertilization, temperature and drought are known to influence the level of ergovaline in endophyte-infected tall fescue [95,101,102,103,104,105,106]. However less is known about the influence of these factors on the concentration of lolitrem B in endophyte-infected perennial ryegrass. Data obtained under controlled conditions revealed that high nitrogen input reduced the levels of endophyte in the plant, which, in turn, reduced alkaloid content [107,108]. A study conducted in field conditions with nitrogen fertilization according to the usual recommendations for pasture suggested that nitrogen input had no effect on the level of lolitrem B in the whole plant, whereas it increased the level of ergovaline [96]. In the same way, little is known about the influence of drought and rainfall on lolitrem B. An analysis of the influence of climatic factors on endophyte-infected perennial ryegrass under field conditions suggested a positive correlation between the cumulative rainfall and the lolitrem B levels in the whole plant [96]. Outbreaks of perennial ryegrass toxicosis in Australia were also most severe during periods of high rainfall in spring and summer, but the concentrations of lolitrem B in the whole plant were no greater than the concentrations recorded earlier in the season [109]. Finally, the stage of maturation of the plant appears to be the most important non-genetic factor responsible for variations in the level of lolitrem B in the plant.

## 5. Indole-Diterpene Alkaloids, Not Only Lolitrem B

**Figure 3:**
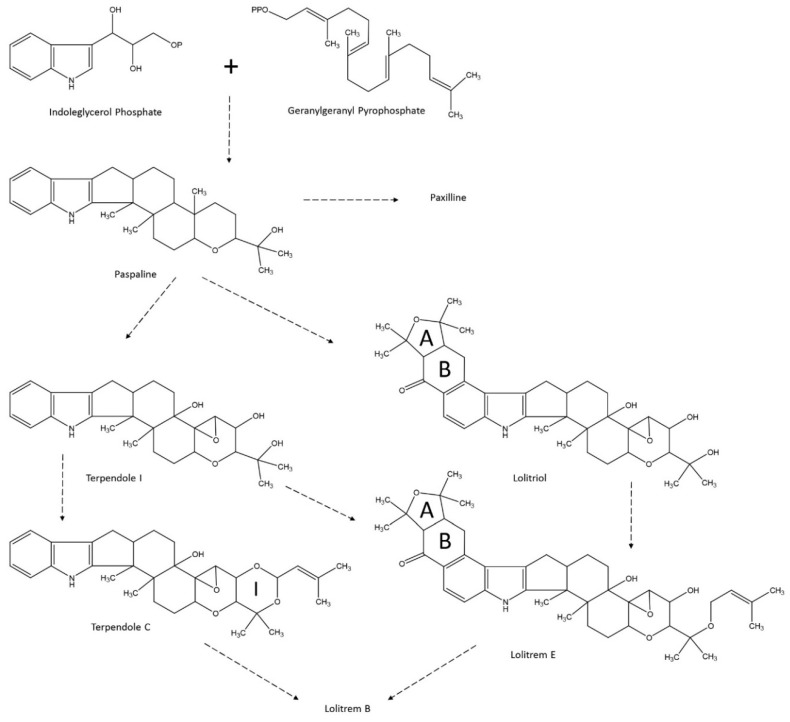
Structures of some intermediaries of synthesis of lolitrem B.

Indole-diterpene alkaloids are formed by a cyclic diterpene-derived skeleton and an indole moiety derived from tryptophan (Figure 3). Different strategies are found in fungal secondary metabolism to incorporate the indole in the final alkaloid metabolites [110]. By analogy with the known pathways for paspaline biosynthesis in *P. janthinellum* and the paxilline biosynthesis in *P. paxilli*, it is suggested that the cyclic diterpene skeleton derived from four isoprene units of geranylgeranyl diphosphate (GGDP), whereas the indole moiety derived from indole-3-gycerophosphate (I3GP) [111]. Paspaline, the simplest indole-diterpene alkaloid, is considered as a key intermediate in the biosynthesis of more complex compounds [58,80]. Paspaline is not tremorgenic. Other indole-diterpenes, such as paspalinine and paspalitrem, also produced by *Claviceps paspalum* in *Paspalum dilatatum* infected seeds, are responsible for paspalum staggers [112,113]. Paspaline can be oxidized into 13-desoxypaxilline then to paxilline by monooxygenases of the cytochrome P450 system [80]. Although paxilline has low tremorgenic properties, few data are available concerning its level in endophyte-infected ryegrass [114,115,116]. Oxidation of paspaline can also produce more than a dozen terpendoles, which are labeled by a different letter (A to M) depending on the number and position of hydroxyl substituents on the diterpene moiety of the molecule. Not all the terpendoles characterized were found in endophyte-infected ryegrass and not all the terpendoles found were tremorgenic. Terpendole I was prenylated and cyclized to form an “I ring” and terpendole C, which was found in endophyte-infected ryegrass and has tremorgenic properties similar to those of paxilline in the mouse bioassay [116].

Lolitrems can be formed from paspaline and terpendoles and other intermediate metabolites by adding an “A” and “B” ring to the indole moiety of the molecule at the C20–C21 position [80]. More than a dozen lolitrems have been characterized and labeled by a letter (A to N). They differ by the presence or absence of an I ring and the number and position of hydroxyl and aryl substituents. The tremorgenic properties of these compounds vary considerably. Although the relationship structure activity of the different lolitrems identified is complex, the presence of the I ring appeared to be necessary to cause prolonged tremors in the mouse bioassay [116]. For example, lolitrem E has no I ring and low tremorgenic properties [117]. Comparison of four stereoisomers of lolitrem B (including lolitrem F, a natural stereoisomer of lolitrem B) also revealed that the stereochemistry of the A/B ring junction does not influence the tremorgenic properties unless the A ring is oriented toward the alpha-face, which stops activity [44]. Lolilline, lolitriol, and lolicines are intermediate metabolites of lolitrems that have an A and B ring on the indole moiety of the molecule but no I ring. These compounds can be found in endophyte-infected ryegrass, but lolilline and lolitriol did not present tremorgenic properties [19,116].

Janthitrems also have an “A” and “B” ring linked to the indole moiety of the molecule, but at the C21–C22 position (Figure 2). These compounds were first isolated from *Penicillium janthinellum* strains obtained from pastures in which ryegrass staggers have been described [118]. Seven janthitrems were characterized and labelled by a letter (A to G). They differ in the number and position of hydroxyl and acetate substitution on the H ring [119]. In contrast to lolitrems, most janthitrems are not epoxidized at the C11–C12 position, but the epoxidized form of janthitrem G was characterized in perennial ryegrass infected with *E. festucae* var. *lolii* strain AR37 [3]. Epoxy-janthitrems can pass through the digestive tract of animals and are found as residues in milk and fat [31,55]. Janthitrems and epoxy-janthitrems have been shown to have tremorgenic properties in the mouse bioassay, but they are less potent than lolitrem B [55,118]. Interestingly, the protection against pest crops observed in perennial ryegrass infected with AR37 is close to that observed with “wild” endophytes [73]. So, despite high concentrations of epoxy-janthitrems found in the fat and milk of animals grazing these pastures, because the signs of staggers were weak and because the protective effect against crop pests were high, it was estimated that the ratio of risk to benefit of AR37 was positive in New Zealand [120].

In conclusion, the symbiotic development of *Epichloë* in perennial ryegrass leads to the production of different groups of alkaloids. Among them, lolitrem B has been shown to be the most toxic alkaloid of the indole diterpene group, responsible for ryegrass staggers, probably because of its binding to the BK channels. A correlation between the rate of infestation and occurrence of the disease has been observed in Australasia, but high rates of endophyte infection of perennial ryegrass with high concentrations of lolitrem B in plants have also been observed worldwide, with only a few atypical cases of staggers outside Australasia. Several factors could explain these differences, such as the use of perennial ryegrass as a monocrop in Australasia and the livestock raising practices. Indeed, the level of lolitrem B in grass varies considerably depending on the stage of maturity of the plant, hence the risk of toxicity varies with the period of the year and the part of the plant consumed. Due to the severity of ryegrass staggers and the high prevalence of crop pests in Australasia, most research has focused on the selection of *Epichloë* unable to produce toxic alkaloids for mammalian herbivores but still able to produce alkaloids that are toxic for insects and nematodes. This target was difficult to achieve. Among the *Epichloë* strains studied, the strains unable to produce lolitrem B and ergovaline but able to produce high levels of epoxy-janthitrems appear to be a good compromise between the risk of staggers in livestock and the need for effective crop pests control in Australasia. Although other indole diterpene alkaloids besides lolitrems and janthitrems were produced by *Epichloë* and have toxic properties, little is known about their level in plants.
